# Response inhibition in problematic social network sites use: an ERP study

**DOI:** 10.3758/s13415-021-00879-9

**Published:** 2021-03-05

**Authors:** Tania Moretta, Giulia Buodo

**Affiliations:** Department of General Psychology, University of Padova, Via Venezia, 12, 35131 Padova, Italy

**Keywords:** Emotional Go/Nogo, Event-related potentials, Inhibitory processes, Internet addiction, Problematic Facebook use

## Abstract

Given the current literature debate on whether or not Problematic Social Network Sites Use (PSNSU) can be considered a behavioral addiction, the present study was designed to test whether, similarly to addictive behaviors, PSNSU is characterized by a deficit in inhibitory control in emotional and addiction-related contexts. Twenty-two problematic Facebook users and 23 nonproblematic users were recruited based on their score on the Problematic Facebook Use Scale. The event-related potentials were recorded during an emotional Go/Nogo Task, including Facebook-related, unpleasant, pleasant, and neutral pictures. The amplitudes of the Nogo-N2 and the Nogo-P3 were computed as measures of the detection of response conflict and response inhibition, respectively. Reaction times and accuracy also were measured. The results showed that problematic users were less accurate on both Go and Nogo trials than nonproblematic users, irrespective of picture content. For problematic users only, the Nogo-P3 amplitude was lower to Facebook-related, pleasant, and neutral than to unpleasant stimuli, suggesting less efficient inhibition with natural and Facebook-related rewards. Of note, all participants were slower to respond to Facebook-related and pleasant Go trials compared with unpleasant and neutral pictures. Consistently, the Nogo-N2 amplitude was larger to Facebook-related than all other picture contents in both groups. Overall, the findings suggest that PSNSU is associated with reduced inhibitory control. These results should be considered in the debate about the neural correlates of PSNSU, suggesting more similarities than differences between PSNSU and addictive behaviors.

**Supplementary Information:**

The online version contains supplementary material available at 10.3758/s13415-021-00879-9.

## Introduction

Although using social networking sites has been described as a potentially addictive behavior (Griffiths, Kuss, & Demetrovics, 2014; Hormes, Kearns, & Timko, 2014; Wang, Sigerson, & Cheng, 2019), the cognitive/affective processes involved in problematic social network sites use remain unclear. One of the key features that have been hypothesized to be at the basis of specific problematic Internet use, including problematic social network sites use, is reduced response-inhibition capacity (Brand, Young, Laier, Wölfling, & Potenza, 2016; Ferraro, Holfeld, Frankl, Frye, & Halvorson, 2015; Luijten et al., 2014). Specifically, in the context of addictive behaviors, it has been argued that compulsivity in engaging in a specific behavior (e.g., using social networking sites, gaming, pornography) may arise from craving symptoms triggered not only by reactivity to addiction-related stimuli but also by defective inhibitory control processes (Brand et al., 2016, 2019; Potenza, 2006). In particular, inhibitory control appears to be impacted adversely by exposure to disorder-related stimuli or highly arousing pleasant and unpleasant stimuli (Bechara, 2003; Moretta, Sarlo, & Buodo, 2019). However, while inhibitory control processes in an emotional context are relatively well-studied in gambling disorder and gaming disorder, much less research has been conducted on other types of behaviors that potentially may become addictive (e.g., social-networking; Brand et al., 2019).

Inhibitory control in emotional contexts can be investigated using the emotional Go/Nogo task, where affective stimuli (e.g., emotionally salient words or pictures) are used in place of standard neutral stimuli, thereby providing a reliable measure of the emotional modulation of behavioral response (Schulz et al., 2007). Two components of the event-related potentials (ERPs), i.e., the Nogo-N2 and the Nogo-P3 (Eimer, 1993; Kiefer, Marzinzik, Weisbrod, Scherg, & Spitzer, 1998), specifically reflect different aspects of response inhibition. The Nogo-N2 is a negative deflection occurring 250-350 ms following Nogo stimuli, with maximum amplitude over frontocentral scalp locations. This component has been suggested to reflect early cognitive control processes necessary to implement inhibitory control, the most important being the detection of conflict between response execution and inhibition (Donkers & Boxtel, 2004; Luijten et al., 2014; Nieuwenhuis, Yeung, Wildenberg, & Ridderinkhof, 2003). The Nogo-P3 is a positive deflection occurring 300-600 ms following Nogo stimuli, with maximum amplitude over frontocentral scalp sites (Kiefer et al., 1998). The Nogo-P3 is thought to reflect successful motor response suppression and/or the evaluation of the outcome of inhibition, and its neural source has been found to be close to the motor and premotor cortices (Bruin, Wijers, & van Staveren, 2001).

In healthy individuals, reaction times (RTs) have been shown to be faster in response to pleasant and unpleasant than neutral Go stimuli, whereas RTs to pleasant and unpleasant Go stimuli were found to be comparable (Chiu, Holmes, & Pizzagalli, 2008) or faster to pleasant than unpleasant Go stimuli (Albert, López-Martín, & Carretié, 2010). There also is evidence that accuracy to Go trials (correct hits) is higher in response to pleasant and unpleasant than neutral conditions (Zhang & Lu, 2012). As for the ERP components, the amplitude of the Nogo-N2 appears not to be modulated by the emotional valence of stimuli. Specifically, no differences in Nogo-N2 amplitudes between emotional and neutral stimuli have been observed (Zhang & Lu, 2012). In contrast, the Nogo-P3 has been shown to be larger in response to emotionally arousing than neutral stimuli, suggesting that the more prepotent the tendency to respond induced by the emotion-laden stimuli, the greater the effort required to inhibit the response (Zhang & Lu, 2012).

In the context of problematic Internet use, ERP studies using the Go/Nogo task highlighted cognitive inefficiency and reduced response-inhibition capacity among individuals with problematic Internet use, as indicated by reduced and enhanced amplitudes of the Nogo-N2 and the Nogo-P3, respectively, among problematic Internet users compared with controls (Dong, Lu, Zhou, & Zhao, 2010; Zhou, Yuan, Yao, Li, & Cheng, 2010). However, the ERP findings of a study on excessive gaming contradict those of the other studies on general problematic Internet use by showing larger Nogo-N2 amplitudes in excessive gamers compared with controls (Littel et al., 2012). To the best of our knowledge, the only study that investigated inhibitory control in problematic social network sites use, in the context of emotionally salient stimuli, employed only disorder-related stimuli as emotionally salient cues (Gao, Jia, Zhao, & Zhang, 2019). In this study, inhibitory control processes were assessed by recording the ERPs during a Go/Nogo task, including social networking sites-related (i.e., WeChat and QQ logos) and neutral images. Despite behavioral measures (RTs to Go trials and/or accuracy) showed no differences between excessive users and controls, ERPs findings highlighted enhanced N2 (to Go and Nogo trials) and reduced Nogo-P3 amplitudes in excessive users as compared with controls, irrespective of stimulus content, suggesting a hypersensitive process of response selection and difficulty in motor inhibition, respectively (Gao et al., 2019). However, it would be important to investigate whether not only the processing of disorder-related stimuli, but also nondisorder-related, highly arousing pleasant and unpleasant stimuli modulates response inhibition in behavioral addictions, and in problematic social network sites use in particular, as it does in substance addiction (Goldstein & Volkow, 2011). Given that a deficit in the modulation of emotional arousal and in the ability to act in desired ways, regardless of emotional state (Gratz & Roemer, 2004), is currently regarded as critically implicated in the development and maintenance of problematic social network sites use (Casale, Caplan, & Fioravanti, 2016; LaRose, Lin, & Eastin, 2003; Moretta & Buodo, 2018; Spada & Marino, 2017; Yu, Kim, & Hay, 2013), the investigation of inhibitory control in emotional contexts that are not only specifically related to social networking sites use would contribute to a better understanding of emotional regulation abilities in problematic social network sites use.

No study to our knowledge has yet investigated whether the processing of social network sites-related and highly arousing emotional stimuli modulates response inhibition in problematic social network sites use (specifically, problematic Facebook use, PFU). In the present study, neural and behavioral measures allowed investigating whether individuals with versus without PFU show greater difficulties in inhibiting prepotent motor responses during an emotional Go/Nogo task.

We expected individuals with PFU to be characterized by impaired inhibitory processes in an emotional context as indicated by faster RTs to Go trials, more commission errors (i.e., responses to Nogo trials), and by larger amplitude of the Nogo-N2 and/or reduced amplitude of the Nogo-P3 in the presence of Facebook-related and emotional versus neutral pictures, and with respect to nonproblematic Facebook users. Also, we hypothesized that individuals with PFU would rate Facebook-related pictures as more pleasant and arousing than neutral pictures and as compared with nonproblematic Facebook users.

## Method

### Participants

Students were contacted informally at university facilities and asked to fill in an online version of the Problematic Facebook Use Scale (PFUS; Marino, Vieno, Altoè, & Spada, 2017). The PFUS is a 15-item scale adapted from the Generalized Problematic Internet Use Scale 2 (Caplan, 2010). In the PFUS, the word “Internet” has been replaced with the word “Facebook” where necessary. The PFUS includes five subscales, i.e., preference for online social interaction, mood regulation, cognitive preoccupation, compulsive use, and negative outcomes. Participants are asked to rate the extent to which they agree with each of the 15 items on an 8-point scale (from 1 = “definitely disagree” to 8 = “definitely agree”). Scores range from 15 to 120; higher scores indicated the presence of relevant Facebook use-related symptomatology. The Italian version of the PFUS has shown a good construct and convergent validity (Marino et al., 2017).

Given that the present study is the first to describe whether the processing of social network sites-related and highly arousing emotional stimuli modulates response inhibition in problematic social network sites use, there was no related effect size to choose from for formal power analysis. The present study has been conducted as a first hypothesis testing and should be used to design larger confirmatory studies. At the beginning, we aimed to recruit about 50 students. In practice, we were able to collect data from 46 participants by the end of the academic year. Data from one participant was excluded due to excessive artifacts (more than 40% of rejected trials).

Based on the scores obtained in the Italian study that validated the PFUS (Marino et al., 2017), 22 participants who scored equal to or higher than 30 (i.e., the 75th percentile) were included in the problematic Facebook users (PFUs) group. Twenty-three participants who scored equal to or lower than 23 (i.e., the 50th percentile) were included in the non-PFUs group. We used scores <50^th^ and >75^th^ percentiles, because the data distribution of the Italian validation of the PFUS was right-skewed with skewness falling before the 50^th^ percentile (thus representative of the lack of relevant Facebook use-related symptomatology). Scores >75^th^ would indicate the presence of relevant Facebook use-related symptomatology.

As reported in Table 1, the two groups differed significantly on PFUS scores and statistically significant differences were not found for sex distribution, age, and sleep hours. All participants read, understood, and signed informed consent. The study was conducted in compliance with the World Medical Association Declaration of Helsinki on research on human subjects and was approved by the Ethical Committee of Psychological Research, Area 17, University of Padova (prot. N. 2312).

**Table 1:** Descriptive statistics and differences between Problematic (PFUs) and nonproblematic (non-PFUs) Facebook users

	Non-PFUs (n = 23)	PFUs (n = 22)	Test-statistic	*p* value
	Mean (±SD)	Mean (±SD)		
PFUS total score	19.0 (±2.3)	49.1 (±15.7)	9.11 ^t test^	<0.001***
Sex (F/M)	20/3	19/3	0.06^†Glm, z test^	0.95
Age	22.3 (±1.5)	22.4 (±2.1)	0. 2^t test^	0.85
Sleep hours per day	7.5 (±0.6)	7.6 (±0.7)	0.6 ^t test^	0.55
Cigarette consumption per day	1.8 (±3.6)	0.8 (±2.3)	−1.15^t test^	0.26
BIS total score	57.8 (±8.7)	61.4 (±8.1)	1.45 ^t test^	0.16

^†^Glm = generalized linear model with binomial error distribution.

### Self-report measure

Given that trait impulsivity, which reflects inhibitory dyscontrol (Enticott, Ogloff, & Bradshaw, 2006; Logan, Schachar, & Tannock, 1997), often has been found to be increased among individuals with problematic Internet use (Rothen et al., 2018), the participants’ trait impulsivity was measured and controlled for in data analysis.

Trait impulsivity was assessed by the Barratt Impulsiveness Scale (BIS-11, Fossati, Ceglie, Acquarini, & Barratt, 2001; Patton, Stanford, & Barratt, 1995). The Italian version of the BIS-11 is a reliable psychometric instrument for measuring impulsiveness (Cronbach’s α = 0.79). It showed good criterion-related validity and has been described as a useful instrument for assessing impulsiveness in nonclinical samples (Fossati et al., 2001). The higher the total score (range = 30–120), the higher the level of trait impulsiveness.

### Emotional Go/Nogo task

The task used in the present study consisted of the presentation of Facebook-related and affective pictures as Go and Nogo stimuli in an emotional Go/Nogo task. A total of 120 (538 × 720 pixels) were presented to each participant, divided into four categories: 30 Facebook-related (copyright-free pictures downloaded from websites, showing devices connected to Facebook. Pictures did not include the entire Facebook user but only his/her hands while using a device connected to Facebook. Comments and/or nicknames appearing in the pictures have been blurred; e.g., Supplementary Figure S1), and 30 pleasant (sport/adventure, erotic couples), 30 unpleasant (attacking humans and animals), and 30 neutral (neutral faces, household objects), selected from the International Affective Picture System (IAPS; Lang, Bradley, & Cuthbert, 2008).^1^ Pleasant and unpleasant pictures were matched for normative Arousal ratings (pleasant = 6.45 ± 2.07; unpleasant = 6.43 ± 2.12; *p* = 1), which were significantly higher than for neutral pictures (neutral = 3.62 ± 1.92; *p*s < 0.001). Pleasant and unpleasant pictures differed significantly for mean normative Valence ratings (pleasant = 6.77 ± 1.82; unpleasant = 2.99 ± 1.73, *p* < 0.001) which were significantly higher and lower, respectively, than for neutral pictures (5.35 ± 1.27; *p*s < 0.001).

Each picture had a pink or blue frame. The color of the frame cued the participant to either press a button (e.g., blue: Go cues) or withhold the response (e.g., pink: Nogo cues). The colors of the frame indicating Go and Nogo cues were counterbalanced across participants. The percentage of Go and Nogo cues was 70% and 30%, respectively, in order to increase the tendency to respond in participants. The 120 pictures were presented five times for a total of 600 trials (420 Go and 180 Nogo). These 600 stimuli were presented in two blocks of 300 trials. The Go and Nogo stimuli were presented for 600 ms in a semirandom sequence (i.e., no more than 2 Nogo stimuli had to be shown consecutively). Each picture (585 × 765 pixels) was preceded by a 500-ms black interval with a white fixation-cross; all the pictures and the fixation cross were placed centrally on the screen. The interstimulus interval was randomly varied between 500 and 800 ms.

The task was programmed using E-Prime software (version 2.0, Psychology Software Tools, Pittsburgh, PA) and was presented by a Core i5-4440 computer on a 19-inch computer screen, at a viewing distance of 1 m.

### Behavioral measures

RTs to Go trials and accuracy in Go and Nogo trials (i.e., keypresses in Go trials and no responses in Nogo trials, respectively) were calculated for each emotional category. Given that RTs below 150 ms can be considered as anticipation errors, they were excluded from the analyses. In the present study, the exclusion of long RTs (e.g., >1,000 ms) was not applied as the longest recorded RT was 412 ms.

### EEG recording

The electroencephalogram (EEG) was recorded using an elastic cap with tin electrodes (ANT Neuro Company), according to the 10–20 System, from 32 scalp positions (i.e., Fp1, Fpz, Fp2, F7, F3, Fz, F4, F8, FC5, FC1, FC2, FC6, T7, C3, Cz, C4, T8, CP5, CP1, CP2, CP6, P7, P3, Pz, P4, P8, POz, O1, Oz, O2, and M1 and M2 [mastoids]), referenced online to Cz.

Both vertical and horizontal electrooculograms (EOGs) were recorded using a bipolar montage to monitor eye movements and eyeblinks. The electrode pairs were placed at the supra- and suborbit of the right eye and at the external canthi of the eyes, respectively. All electrophysiological signals were amplified with a EEGO amplifier (ANT Neuro Company, https://www.ant-neuro.com/products/eego_mylab). All electrode impedances were kept below 5 kΩ. The EEG signal was bandpass filtered online (0.1–40 Hz) and digitized at 1,000 Hz. Offline, the EEG was re-referenced to mastoids, corrected for eyeblink artifacts using independent component analysis, and low-pass filtered at 30 Hz.

Filtering and further EEG processing were run in Brain Vision Analyzer 2.1 software. EEG epochs of −100 to 600 ms post-stimulus were baseline-corrected by subtracting the mean voltage during the 100-ms prestimulus period and segments containing residual artifacts exceeding ±70 μV (peak-to-peak) were excluded. By applying the a priori criteria of excluding individuals for whom more than 40% of trials were rejected, one participant in the PFUs group was excluded. The corrected EEG epochs were averaged separately for each participant and experimental condition. Individual ERP averages were derived for correct Go and Nogo trials (i.e., excluding Go trials with missed responses and Nogo trials with commission errors). According to the literature (Falkenstein, Hoormann, & Hohnsbein, 1999) and to visual inspection of the grand-average ERP waveforms at frontocentral electrodes (F3, Fz, F4, C3, Cz, C4), where N2 and P3 amplitudes reach their maximum on response inhibition tasks (Falkenstein et al., 1999), the mean amplitudes of the following ERP components were computed: N2, as the mean amplitude 220-280 ms after stimulus onset; P3, as the mean amplitude 340-420 ms after stimulus onset. Moreover, based on the inspection of grand-average ERP waveforms, P2 also was computed as the mean amplitude of 160-210 ms after stimulus onset.

### Procedure

Upon arrival at the laboratory, the participants read and signed an informed consent form and were seated in a comfortable armchair in a sound-attenuated, dimly lit room. Then, each participant completed the BIS-11. After the electrodes were attached, the participants were instructed to press a key with the index finger of their right hand, as rapidly and accurately as possible, when a picture with the Go color frame (e.g., pink) was presented and to withhold pressing the key upon the presentation of a picture with the Nogo color frame (e.g., blue). Before the beginning of the task, participants underwent a practice block of 10 trials (7 Go and 3 Nogo), to ensure they understood task instructions. The participants were also asked to maintain their gaze on the fixation cross. Each participant was allowed to rest between the two experimental blocks.

After the experimental session, the participants performed Valence and Arousal ratings for all pictures used in the emotional Go/Nogo task, using a computerized version of the 1–9 point scales of Valence and Arousal of the Self-Assessment Manikin (SAM; Bradley & Lang, 1994).

### Statistical analysis

All analyses were performed using R software (R Development Core Team, 2016). As a statistically significant difference in BIS-11 total scores did not emerge between groups (*Cohen d* = 0.44; Table 1), impulsivity was not included as a covariate in the analyses.

To investigate whether the two groups differed in terms of response accuracy to Go and Nogo trials, we estimated a generalized linear mixed-effects model (GLMM) with binomial error distribution and individuals as a random term. The GLMM included Condition (Go, Nogo), Category (Facebook-related, Pleasant, Unpleasant, and Neutral), Group (PFUs, non-PFUs), and their interactions as fixed factors.

Before running the other analyses, all data were examined for skewness, kurtosis, outliers, and normalcy by both exploratory analyses and graphs, i.e., violin plots and boxplots (Pastore, Lionetti, & Altoè, 2017). The normal Probability-Probability plot of the standardized residuals showed points that were close on the line, and the scatterplot of the standardized residuals showed that the data met the assumptions of homogeneity of variance and linearity for all dependent variables. Thus, to compare RTs to Go trials between groups, a linear mixed-effect model (LMM) with individual random intercept (R package: lme4, Bates, Maechler, Bolker, & Walker, 2014) was conducted on RTs to Go trials with Category (i.e., Facebook-related, Pleasant, Unpleasant, and Neutral), and Group (PFUs, non-PFUs) as fixed factors.

As for the analysis of ERP data, in a first step, the effect of Condition (Go, Nogo) on both N2 and P3 amplitudes was checked by a linear mixed-effect model (LMM) with individual random intercept and Condition as a fixed factor. Then, LMMs with individual random intercept were conducted on the mean amplitudes of Nogo-N2 and Nogo-P3 components, with Group (PFU, non-PFU), Category (Facebook-related, Pleasant, Unpleasant, and Neutral), and their interaction as fixed factors.^2^ ERP analysis was focused on Nogo trials given that only Nogo-N2 and Nogo-P3 amplitudes reflect the inhibitory processes and therefore are directly relevant for the research question addressed in the present study.

For exploratory purposes, an LMM with individual random intercept was also conducted on the mean amplitudes of Nogo-P2 component, with Group (PFU, non-PFU), Category (Facebook-related, Pleasant, Unpleasant, and Neutral), and their interaction as fixed factors.^2^

Valence and Arousal ratings were submitted to separate LMMs, with individuals and pictures as random terms, and Category (Facebook-related, Pleasant, Unpleasant, and Neutral) and Group (PFUs, non-PFUs) as fixed factors.

Overall, the strength of parameters evidence within the models was estimated as the difference in the Akaike information criterion (AIC) between the model without and the model with the parameter (ΔAIC, Wagenmakers & Farrell, 2004; Burnham & Anderson, 2002). Denominator degrees of freedom for F-tests were estimated by Satterthwaite and Kenward-Roger methods (Kuznetsova, Brockhoff, & Christensen, 2017), and Bonferroni HSD post-hoc tests were employed to further examine significant effects (using a *p* < 0.05 criterion for significance).

Lastly, Pearson’s correlation coefficients were calculated between Nogo-N2 and Nogo-P3 amplitudes at frontal and central midline sites (Fz, Cz), behavioral measures (RTs to Go trials, accuracy in Nogo trials), and Valence and Arousal ratings separately for PFUs and non-PFUs. To correct for type I error rate (false–positive correlations), Bonferroni correction for multiple testing was applied and the alpha significance level was set on *p* < 0.003. Only statistically significant results have been reported.

## Results

Descriptive statistics are reported in Table 1.

### Behavioral data

#### Accuracy to Go and Nogo trials

A statistically significant effect of Condition was found (χ^2^_1_ = 632.23, *p* < 0.001, ΔAIC = 14499, odds ratio [OR] = 31.7), indicating that accuracy was lower for Nogo (mean = 90.35%, SD = 8.34) than for Go trials (mean = 99.76%, SD = 0.48).

A statistically significant main effect of Category also was found (χ^2^_3_ = 17.86, *p* < 0.001, ΔAIC = 4775); however, post-hoc comparisons did not reveal significant differences between emotional categories (Facebook-related: mean = 94.62%, SD = 8.33; Pleasant: mean = 94.40%, SD = 7.84; Unpleasant: mean = 96.16%, SD = 6.12; Neutral: mean = 95.04%, SD = 7.63).

The statistically significant main effect of Group (χ^2^_1_ = 4.43, P = .03, ΔAIC = 4674, OR = 1.5) showed that PFU were likelier to be significantly less accurate (mean = 94.23, SD = 8.12) than non-PFU (mean = 95.84, SD = 6.87).

#### Reaction times (RTs) to Go trials

Only a statistically significant main effect of Category was found (χ^2^_3_ = 27.29, *p* < 0.001, ΔAIC = 37.5). Overall, participants were slower in the presence of both Pleasant (mean = 347.16, SD = 30.58) and Facebook-related (mean = 346.65, SD = 29.63) than Neutral (mean = 339.82, SD = 28.55) and Unpleasant (mean = 340.85, SD = 27.62) pictures (*p*s < 0.01).

### ERP data

**Fig. 1:**
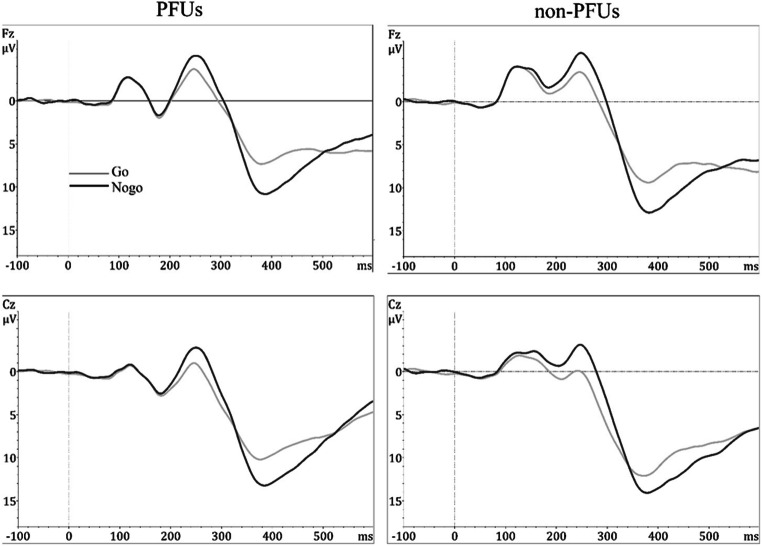
Grand average ERP waveforms recorded at Fz and Cz sites to Go and Nogo trials in PFUs and non-PFUs

A statistically significant effect of Condition was found for both N2 (F_1, 2114_ = 299, *p* < 0.001, ΔAIC = 275) and P3 (F_1, 2114_ = 245, *p* < 0.001, ΔAIC = 228) amplitudes, indicating larger amplitudes for Nogo (N2: mean = −2.69, SD = 3.68; P3: mean = 10.41, SD = 5.09) than Go (N2: mean = −0.65, SD = 3.54; P3: mean = 8.52, SD = 4.09) trials (Fig. 1).

#### Nogo-N2 amplitude

A statistically significant main effect of Category (F_3, 1029_ = 33.08, *p* < 0.001, ΔAIC = 81.6) was found, highlighting larger Nogo-N2 amplitude to Facebook-related (mean = −3.88 μV, SD = 3.92) than to Neutral (mean = −2.77 μV, SD = 3.38), Unpleasant (mean = −1.74 μV, SD = 3.50), and Pleasant pictures (mean = −2.36 μV, SD = 3.59, all *p*s < 0.001). Nogo-N2 amplitude was smaller to Unpleasant than to Facebook-related, Neutral (both *p*s < 0.001), and Pleasant stimuli (*p* = 0.05). The Nogo-N2 amplitudes to Pleasant and Neutral stimuli did not differ significantly from each other.

#### Nogo-P3 amplitude

A statistically significant main effect of Category (F_3,1029_ = 13.82, *p* < 0.001, ΔAIC = 35.7) was found, highlighting larger Nogo-P3 amplitude to Facebook-related (mean = 10.50 μV, SD = 5.35), Unpleasant (mean = 11.06 μV, SD = 4.94), and Pleasant stimuli (mean = 10.52 μV, SD = 5.05) than to Neutral (mean = 9.57 μV, SD = 4.92, all *p*s < 0.001) in both PFUs and non-PFUs. No significant differences emerged between the Nogo-P3 amplitudes to Facebook-related, Unpleasant, and Pleasant stimuli.

**Fig. 2:**
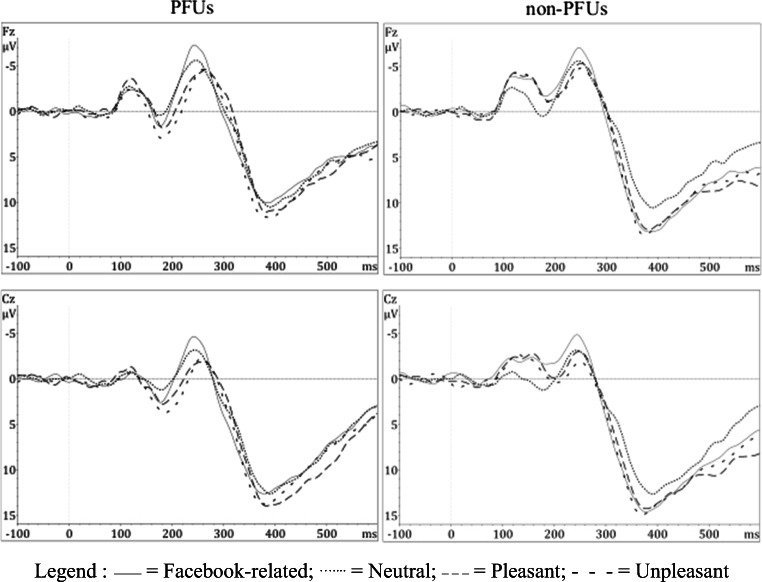
Grand average ERP waveforms recorded at Fz and Cz sites to Nogo trials for Neutral, Facebook-related, Pleasant, and Unpleasant conditions in PFUs and non-PFUs

The statistically significant Group × Category interaction (F_3, 1029_ = 3.5, *p* = 0.015, ΔAIC = 5) showed that in non-PFUs, the amplitude of the Nogo-P3 was larger for Facebook-related (mean = 11.54 μV, SD = 6.27), Unpleasant (mean = 11.59 μV, SD = 5.49), and Pleasant (mean = 11.07 μV, SD = 5.51) than Neutral (mean = 9.89 μV, SD = 5.48. all *p*s < 0.01) pictures. The Nogo-P3 amplitudes to Facebook-related, Pleasant, and Unpleasant pictures did not differ significantly from each other. Differently, in PFUs, the Nogo-P3 amplitudes to Neutral (mean = 9.24 μV, SD = 4.25), Facebook-related (mean = 9.42 μV, SD = 3.92), and Pleasant (mean = 9.95 μV, SD = 4.46) pictures were comparable. The Nogo-P3 amplitude was larger to Unpleasant (mean = 10.51 μV, SD = 4.25) than to Facebook-related and Neutral pictures (Fig. 2).

#### Exploratory analysis: Nogo-P2 amplitude

A statistically significant main effect of Group (F_3,1029_ = 15.45, *p* < 0.001, ΔAIC = 16.5) was found, highlighting larger Nogo-P2 amplitude in PFUs (mean = 1.08 μV, SD = 3.02) than to non-PFUs (mean = −1.64 μV, SD = 3.01) to all picture contents.

A statistically significant main effect of Category (F_3,1029_ = 39.76, *p* < 0.001, ΔAIC = 103) also was found with larger Nogo-P2 amplitude to Unpleasant (mean = 0.61 μV, SD = 3.40) than to Facebook-related (mean = −0.67 μV, SD = 3.43), Pleasant (mean = -0.11 μV, SD = 3.19), and Neutral stimuli (mean = −1.06 μV, SD = 2.95, all *p*s < 0.001). Moreover, we found larger Nogo-P2 amplitude to Pleasant than Neutral and Facebook-related stimuli (*p*s < 0.01). No significant differences emerged between the Nogo-P2 amplitudes to Facebook-related and Neutral stimuli.

These effects were specified by the statistically significant Group × Category interaction (F_3, 1029_ = 3.94, *p* = 0.008, ΔAIC = 4) showing that in non-PFUs, the amplitude of the Nogo-P2 was larger for Unpleasant (mean = −1.02 μV, SD = 2.81) than Facebook-related (mean = −2.00 μV, SD = 3.47) and Neutral pictures (mean = −2.15 μV, SD = 2.76. All *p*s < 0.01) and for Pleasant (mean = −1.39 μV, SD = 2.81) than Neutral pictures (*p* = 0.01). Differently, in PFUs, the Nogo-P2 amplitude was larger for Unpleasant (mean = 2.32 μV, SD = 3.12) than Pleasant (mean = 1.22 μV, SD = 3.02), Facebook-related (mean = 0.72 μV, SD = 2.78), and Neutral pictures (mean = 0.08 μV, SD = 2.72. All *p*s < 0.001) and for Pleasant (mean = 1.22 μV, SD = 3.02) than Neutral pictures (*p* < 0.001). Between-group differences were also found, with larger Nogo-P2 amplitude to Unpleasant, Pleasant, and Facebook-related pictures in PFUs than non-PFUs (all *p*s < 0.05).

### Valence and Arousal ratings

The Group main effect was statistically significant only for Arousal ratings (F_1,43_ = 6.78, *p* = 0.01, ΔAIC = 198). Regardless of their emotional category, pictures were rated as more arousing for PFUs than non-PFUs.

The Category main effect was statistically significant for both Valence and Arousal ratings (Valence: F_3,116_ = 355.26, *p* < 0.001, ΔAIC = 321; Arousal: F_3,116_ = 518.74, *p* < 0.001, ΔAIC = 496). Unpleasant pictures were rated as significantly more arousing and unpleasant than all other picture categories (all *p*s < 0.001). Moreover, pleasant pictures were rated as significantly more arousing than Facebook-related and Neutral pictures and more pleasant than all other picture categories (all *p*s < 0.001). Facebook-related pictures were rated as more arousing than Neutral pictures (*p* = 0.001). As for Valence ratings, no difference was found between Neutral and Facebook-related pictures.

**Fig. 3:**
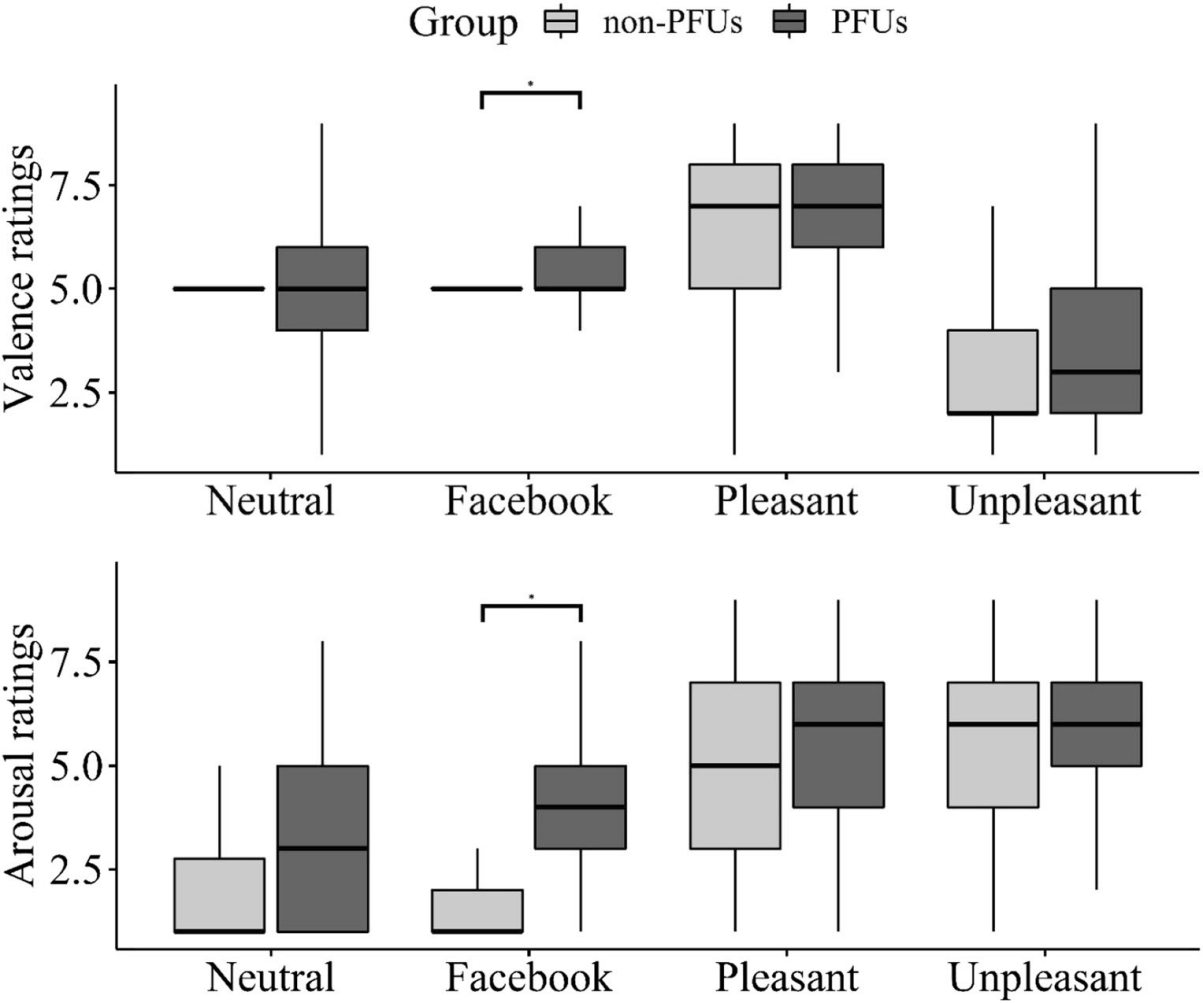
Valence and Arousal ratings in non-PFU vs. PFU. Asterisks and lines indicate statistically significant differences between non-PFU and PFU

These effects were specified by the statistically significant Group × Category interactions (arousal: F_3, 5233_ = 70.53, *p* < 0.001, ΔAIC = 193; valence: F_3, 5233_ = 24.57, *p* < 0.001, ΔAIC = 58.8). As shown in Fig. 3, in both groups Unpleasant pictures elicited significantly greater unpleasantness and arousal than all other picture categories (all *p*s < 0.01), and Pleasant pictures elicited significantly greater pleasantness and greater arousal than Neutral and Facebook-related pictures (all *p*s < 0.001). In non-PFUs, no differences between Neutral and Facebook-related pictures were found for Arousal and Valence ratings. Conversely, PFUs rated Facebook-related pictures as significantly more pleasant and arousing than Neutral pictures (*p* < 0.001). As for between-group differences, PFUs rated Facebook-related pictures as significantly more arousing and pleasant than non-PFUs (both *p*s < 0.05). No between-group differences were found for the other emotional categories.

### Correlations between Nogo-ERPs, behavioral data, and subjective ratings

As reported in Table 2, in non-PFUs, the amplitude of the Nogo-N2 for Facebook-related pictures (in Cz) was positively correlated with Arousal ratings. In PFUs, no meaningful correlations were found between Nogo-ERPs, behavioral data, and subjective ratings.

**Table 2:** Pearson’s (r) correlation coefficients between frontal and central (Fz, Cz) Nogo-N2 and Nogo-P3 amplitudes, behavioral data (RTs to Go trials and accuracy in Nogo trials), and subjective ratings (Arousal and Valence) for each emotional category in problematic (PFUs) and nonproblematic (non-PFUs) Facebook users

	PFUs															
	Facebook-related				Neutral				Pleasant				Unpleasant			
	Nogo-N2		Nogo-P3		Nogo-N2		Nogo-P3		Nogo-N2		Nogo-P3		Nogo-N2		Nogo-P3	
	Fz	Cz	Fz	Cz	Fz	Cz	Fz	Cz	Fz	Cz	Fz	Cz	Fz	Cz	Fz	Cz
RTs (ms)^†^	0.11	0.04	−0.08	−0.20	−0.32	−0.35	−0.34	−0.41	−0.29	−0.17	−0.16	−0.11	−0.01	−0.00	−0.25	−0.34
Accuracy (%)^‡^	−0.07	−0.29	0.27	−0.01	−0.03	−0.17	−0.24	−0.08	−0.23	−0.39	−0.10	−0.20	−0.03	−0.17	−0.04	−0.07
Arousal	0.10	0.01	0.33	0.30	−0.13	−0.11	−0.31	−0.20	−0.31	−0.35	−0.12	−0.02	−0.19	−0.13	0.35	0.34
Valence	0.08	0.01	0.24	0.12	−0.33	−0.26	−0.02	0.01	−0.05	−0.06	0.10	0.08	0.14	−0.03	−0.09	-0.12
	**non-PFUs**															
	**Facebook-related**				**Neutral**				**Pleasant**				**Unpleasant**			
	Nogo-N2		Nogo-P3		Nogo-N2		Nogo-P3		Nogo-N2		Nogo-P3		Nogo-N2		Nogo-P3	
	Fz	Cz	Fz	Cz	Fz	Cz	Fz	Cz	Fz	Cz	Fz	Cz	Fz	Cz	Fz	Cz
RTs (ms)^†^	−0.16	−0.19	−0.18	−0.11	−0.36	−0.35	−0.29	−0.26	−0.14	−0.13	−0.28	−0.21	0.04	−0.06	−0.27	-0.24
Accuracy (%)^‡^	0.17	0.32	0.15	0.16	0.25	0.34	0.32	0.22	−0.14	−0.07	−0.43	−0.42	0.03	0.20	−0.15	-0.10
Arousal	0.56	0.62^**^	0.43	0.34	0.19	0.32	0.15	0.04	0.35	0.44	0.09	0.11	0.18	0.44	−0.01	0.05
Valence	0.48	0.55	0.20	0.24	0.17	0.33	0.27	0.20	0.42	0.42	−0.10	−0.17	0.00	−0.17	0.08	0.04

^†^Reaction times to Go trials; ^‡^Percentage of accuracy to Nogo trials.

## Discussion

The present study examined whether the processing of Facebook-related and highly arousing emotional stimuli modulates response inhibition in problematic Facebook users during an emotional Go/Nogo task. It was hypothesized that problematic relative to nonproblematic users would show faster RTs to Go trials, more commission errors in Nogo trials, larger amplitude of the Nogo-N2, and/or reduced amplitude of the Nogo-P3 in the presence of Facebook-related and emotional versus neutral pictures.

Some interesting between- and within-group differences emerged on the behavioral and the neural level, respectively. On the behavioral level, accuracy was lower in problematic versus nonproblematic Facebook users, both when they had to respond to Go stimuli and when they had to withhold from responding to Nogo stimuli, irrespective of the pictures’ content. This seems to suggest an overall greater difficulty with adjusting behavior to contextual demands. Our findings are consistent with those of Zhou et al. (2010) and Moretta et al. (2019), reporting both higher false-alarm and higher miss rates in problematic versus nonproblematic Internet users. Other studies that used a standard Go/Nogo task to investigate inhibitory control in individuals with nonspecific problematic Internet use (Ding et al., 2014; Dong et al., 2010; Sun et al., 2009) did not report reduced performance accuracy in either Go or Nogo trials in excessive versus casual Internet users. No difference in the behavioral performance between excessive social network sites users and controls also was reported by Gao et al. (2019), who used an emotional Go/Nogo task, including social networking sites-related and neutral stimuli, to assess inhibitory control in excessive social network sites users (Gao et al., 2019). It may be hypothesized that similar to that reported for nonspecific problematic Internet use, difficulties in inhibitory control only emerge in problematic Facebook users when the effort required to suppress inappropriate responses exceeds a certain threshold, i.e., when prepotent responses must be inhibited according to complex rules (Zhou et al., 2010), or in an emotional context (Moretta et al., 2019).

On the neural level, only in nonproblematic Facebook users the Nogo-P3 amplitude was modulated by emotional contents, i.e., it was larger to Facebook-related and emotional stimuli versus neutral stimuli., suggesting that Facebook-related and highly arousing emotional stimuli activated similar (successful) inhibitory processes. Interestingly, in problematic Facebook users the Nogo-P3 amplitude was lower to Facebook-related, pleasant, and neutral stimuli than to unpleasant stimuli, suggesting less efficient evaluation of the outcome of inhibition when it is required in the presence of both natural and secondary (Facebook-related) rewards. The Nogo-P3 is taken to reflect the closure of the inhibition process after the decision (Gajewski & Falkenstein, 2013), the evaluation of the inhibitory performance (Bruin et al., 2001; Roche, Garavan, Foxe, & O’Mara, 2005) or the effectiveness of motor inhibition engaged in or near the motor or premotor cortices (Kok, Ramautar, De Ruiter, Band, & Ridderinkhof, 2004; Ramautar, Kok, & Ridderinkhof, 2004).

In contrast, in problematic Facebook users the Nogo-P3 to pleasant, Facebook-related, and neutral did not differ from each other and was significantly reduced compared with the Nogo-P3 to unpleasant pictures. Reduced Nogo-P3 amplitude is considered a robust finding in substance use disorders (Cohen, Porjesz, Begleiter, & Wang, 1997; Colrain et al., 2011). Indeed, it also has been reported in nicotine use disorder (Evans, Park, Maxfield, & Drobes, 2009), alcoholism (Porjesz & Begleiter, 2003), and stimulant use disorder (Sokhadze, Stewart, Hollifield, & Tasman, 2008). Taken together, our findings suggest that similar to substance use disorders, problematic Facebook use is characterized by underengagement of response inhibition processes in the context of natural reward- and Facebook-related stimuli.

Unexpectedly, the exploratory analysis revealed that the amplitude of the Nogo-P2 in response to Facebook-related and affective (pleasant and unpleasant) pictures was significantly larger in problematic as compared to nonproblematic Facebook users, whereas the Nogo-P2 amplitude to neutral pictures did not differ between groups. These results may indicate that in early, automatic stages of information processing, problematic Facebook users already tend to allocate more attention toward Facebook-related and affective stimuli. This result is similar to findings from previous studies that investigated differences in the attentional processing of food-related words in obese and normal-weight individuals (Nijs, Franken, & Muris, 2010).

As expected, problematic users rated Facebook-related pictures as more pleasant and arousing as compared with nonproblematic users, suggesting that similar to drug-related cues in substance use disorders (Engelmann, Gewirtz, & Cuthbert, 2011; Littel & Franken, 2007; Lubman et al., 2009; Wölfling et al., 2011), positive reinforcement from Facebook use may be transferred to Facebook-related stimuli (Everitt et al., 1999; Grimm, 2000).

Of note, Facebook-related pictures were found to modulate inhibitory processes in both problematic and nonproblematic users. Specifically, in the presence of both Facebook-related and pleasant Go trials, RTs were slower compared with unpleasant and neutral pictures, suggesting that in Facebook users, regardless of whether they engage in problematic Facebook-related behavior, Facebook-related stimuli capture attention as much as natural rewards (i.e., sex). Consistently, the Nogo-N2 amplitude was larger to Facebook-related than all other picture contents, suggesting that greater response conflict was generated when Nogo stimuli required to withhold responding in the context of Facebook-related stimuli. In the past decade, it has become increasingly clear that the Nogo-N2 does not properly reflect response inhibition or only to a limited extent (Bruin et al., 2001; Donkers & Boxtel, 2004). Instead, Nogo-N2 is now more commonly regarded as reflecting conflict monitoring performed by the anterior cingulate cortex (ACC; Bekker, Kenemans, & Verbaten, 2005Donkers & Boxtel, 2004; Palermo, Stanziano, & Morese, 2018). In the present study, the lack of significant correlations between performance measures and Nogo-N2 amplitudes for all picture categories in both groups suggests that the neural representation of response conflict was not associated with attentional and motivational aspects of responding (as indexed by RTs to Go trials) and motor inhibition (as indexed by accuracy to Nogo trials). Rather, it may be hypothesized that Facebook-related stimuli trigger higher conflict monitoring performed by the ACC than other affective stimuli, possibly reflecting the potential addictive properties of social network sites (e.g., learned associations between PFU-related stimuli and a pleasurable or an intensely overpowering experience). Future studies are needed to further understand the relationship between the Nogo-N2 amplitude to Facebook (and other social network sites)-related cues and its possible role as a precursor/risk factor for the development of problematic social network sites use.

The present results should be interpreted taking four main limitations into account. The first is the small sample size. Small sample size does not allow precise estimates and larger confirmatory studies are needed. The second is the criteria employed for sample selection. In the present study, participants were classified as problematic Facebook users based on PFUS scores and, unlike participants who received a diagnosis in substance use disorders and behavioral addiction studies, they may not be fully representative of severe, clinically relevant Facebook-related behaviors. Indeed, high scores on the PFUS may reflect at-risk problematic Facebook use. Third, previous studies showed sex differences in the use of social network sites (Mazman & Usluel, 2011). Due to the within-group difference in sex distribution, we were unable to investigate sex differences. Further research, including larger samples and equal sex distributions within-group, should be undertaken. Lastly, our interpretation of ERP results requires caution as, to the best of our knowledge, there are no published ERP studies that used the emotional Go/Nogo task to study inhibitory processes to Facebook-related and affective stimuli in problematic and nonproblematic Facebook users. Further studies that replicate our findings in larger groups of participants are needed to confirm our results.

Overall, our findings suggest that problematic Facebook users are characterized by underengagement of response inhibition processes in the context of natural reward- and Facebook-related stimuli, as indexed by reduced overall accuracy ratings and Nogo-P3 amplitude to Facebook-related and pleasant stimuli. Moreover, problematic users rated Facebook-related pictures as more pleasant and arousing than controls, suggesting that Facebook-related stimuli acquire positive reinforcement properties from Facebook use. Of note, all participants (problematic and non-problematic Facebook users) were slower to respond to both Facebook-related and pleasant Go trials compared with unpleasant and neutral pictures. Consistently, the Nogo-N2 amplitude was larger to Facebook-related than all other picture contents, suggesting that greater response conflict was generated when Nogo stimuli required to withhold responding in the context of Facebook-related stimuli.

## Supplementary Information

ESM 1 (DOCX 750 kb)

## Footnotes

1 The IAPS picture numbers were as follows: Pleasant: 4611, 4647, 4651, 4652, 4658, 4660, 4664, 4670, 4672, 4680, 4683, 4690, 4695, 4800, 4810, 8030, 8031, 8034, 8080, 8160, 8161, 8178, 8179, 8180, 8185, 8186, 8200, 8370, 8400, 8490. Unpleasant: 1050, 1051, 1114, 1120, 1300, 1301, 1302, 1321, 1930, 1932, 3500, 6200, 6210, 6230, 6242, 6243, 6244, 6250, 6260, 6300, 6312, 6313, 6370, 6510, 6540, 6550, 6560, 6571, 6821, 9425. Neutral: 7000, 7002, 7004, 7009, 7010, 7020, 7035, 7036, 7037, 7041, 7050, 7056, 7059, 7130, 7140, 7175, 7217,7224, 7233, 7235, 7242, 7491, 7500, 7546, 7547, 7560, 7590, 7595, 7700, 7950.

2 The results on the Group, Category, and Group×Category did not change if the analyses were re-run by including the Area (frontal [Fz, F3, F4], and central [Cz, C3, C4]) and Laterality (left [F3, C3], midline [Fz, Cz], right [F4, C4]) as fixed factors.

## References

[CR1] Albert J, López-Martín S, Carretié L (2010). Emotional context modulates response inhibition: Neural and behavioral data. NeuroImage.

[CR2] Bates, D., Maechler, M., Bolker, B., & Walker, S. (2014). lme4: Linear mixed-effects models using Eigen and S4. R package version 1.1-7, http://CRAN.R-project.org/package=lme4. *R Package Version*.

[CR3] Bechara A (2003). Risky business: emotion, decision-making, and addiction. Journal of Gambling Studies.

[CR4] Bekker EM, Kenemans JL, Verbaten MN (2005). Source analysis of the N2 in a cued Go/NoGo task. Cognitive Brain Research.

[CR5] Bradley MM, Lang PJ (1994). Measuring emotion: The self-assessment manikin and the semantic differential. Journal of Behavior Therapy and Experimental Psychiatry.

[CR6] Brand M, Wegmann E, Stark R, Müller A, Wölfling K, Robbins TW, Potenza MN (2019). The Interaction of Person-Affect-Cognition-Execution (I-PACE) model for addictive behaviors: Update, generalization to addictive behaviors beyond internet-use disorders, and specification of the process character of addictive behaviors. Neuroscience & Biobehavioral Reviews.

[CR7] Brand M, Young KS, Laier C, Wölfling K, Potenza MN (2016). Integrating psychological and neurobiological considerations regarding the development and maintenance of specific Internet-use disorders: An Interaction of Person-Affect-Cognition-Execution (I-PACE) model. Neuroscience & Biobehavioral Reviews.

[CR8] Bruin K, Wijers A, van Staveren AS (2001). Response priming in a go/nogo task: do we have to explain the go/nogo N2 effect in terms of response activation instead of inhibition?. Clinical Neurophysiology.

[CR9] Burnham, K. P., & Anderson, D. R. (2002). Model selection and multimodel inference: a practical information-theoretic approach, Second Edition. In *Book*.

[CR10] Caplan SE (2010). Theory and measurement of generalized problematic Internet use: A two-step approach. Computers in Human Behavior.

[CR11] Casale S, Caplan SE, Fioravanti G (2016). Positive metacognitions about Internet use: The mediating role in the relationship between emotional dysregulation and problematic use. Addictive Behaviors.

[CR12] Chiu PH, Holmes AJ, Pizzagalli DA (2008). Dissociable recruitment of rostral anterior cingulate and inferior frontal cortex in emotional response inhibition. NeuroImage.

[CR13] Cohen HL, Porjesz B, Begleiter H, Wang W (1997). Neuroelectric correlates of response production and inhibition in individuals at risk to develop alcoholism. Biological Psychiatry.

[CR14] Colrain IM, Sullivan EV, Ford JM, Mathalon DH, McPherson S-L, Roach BJ, Crowley KE, Pfefferbaum A (2011). Frontally mediated inhibitory processing and white matter microstructure: age and alcoholism effects. Psychopharmacology.

[CR15] Ding W, Sun J, Sun Y, Chen X, Zhou Y, Zhuang Z, Li L, Zhang Y, Xu J, Du Y (2014). Trait impulsivity and impaired prefrontal impulse inhibition function in adolescents with internet gaming addiction revealed by a Go/No-Go fMRI study. Behavioral and Brain Functions.

[CR16] Dong G, Lu Q, Zhou H, Zhao X (2010). Impulse inhibition in people with Internet addiction disorder: Electrophysiological evidence from a Go/NoGo study. Neuroscience Letters.

[CR17] Donkers, F. C. L., & van Boxtel, G. J. M. (2004). The N2 in go/no-go tasks reflects conflict monitoring not response inhibition. *Brain and Cognition*, *56*(2), 165–176. 10.1016/j.bandc.2004.04.005 10.1016/j.bandc.2004.04.005 15518933

[CR18] Eimer M (1993). Effects of attention and stimulus probability on ERPs in a Go/Nogo task. Biological Psychology.

[CR19] Engelmann JM, Gewirtz JC, Cuthbert BN (2011). Emotional reactivity to emotional and smoking cues during smoking abstinence: Potentiated startle and P300 suppression. Psychophysiology.

[CR20] Enticott PG, Ogloff JRP, Bradshaw JL (2006). Associations between laboratory measures of executive inhibitory control and self-reported impulsivity. Personality and Individual Differences.

[CR21] Evans DE, Park JY, Maxfield N, Drobes DJ (2009). Neurocognitive variation in smoking behavior and withdrawal: genetic and affective moderators. Genes, Brain and Behavior.

[CR22] Everitt BJ, Parkinson JA, Olmstead MC, Arroyo M, Robledo P, Robbins TW (1999). Associative Processes in Addiction and Reward The Role of Amygdala-Ventral Striatal Subsystems. Annals of the New York Academy of Sciences.

[CR23] Falkenstein, M., Hoormann, J., & Hohnsbein, J. (1999). ERP components in Go/Nogo tasks and their relation to inhibition. *Acta Psychologica*. 10.1016/s0001-6918(99)00008-6 10344188

[CR24] Ferraro FR, Holfeld B, Frankl S, Frye N, Halvorson N (2015). Texting/iPod dependence, executive function and sleep quality in college students. Computers in Human Behavior.

[CR25] Fossati, A., Di Ceglie, A., Acquarini, E., & Barratt, E. S. (2001). Psychometric properties of an Italian version of the Barratt Impulsiveness Scale-11 (BIS-11) in nonclinical subjects. *Journal of Clinical Psychology*, *57*(6), 815–828. 10.1002/jclp.1051 10.1002/jclp.1051 11344467

[CR26] Gajewski PD, Falkenstein M (2013). Effects of task complexity on ERP components in Go/Nogo tasks. International Journal of Psychophysiology.

[CR27] Gao Q, Jia G, Zhao J, Zhang D (2019). Inhibitory Control in Excessive Social Networking Users: Evidence From an Event-Related Potential-Based Go-Nogo Task. Frontiers in Psychology.

[CR28] Goldstein RZ, Volkow ND (2011). Dysfunction of the prefrontal cortex in addiction: neuroimaging findings and clinical implications. Nature Reviews Neuroscience.

[CR29] Gratz KL, Roemer L (2004). Multidimensional Assessment of Emotion Regulation and Dysregulation: Development, Factor Structure, and Initial Validation of the Difficulties in Emotion Regulation Scale. Journal of Psychopathology and Behavioral Assessment.

[CR30] Griffiths, M. D., Kuss, D. J., & Demetrovics, Z. (2014). Social Networking Addiction. In *Behavioral Addictions* (pp. 119–141). Elsevier. 10.1016/B978-0-12-407724-9.00006-9

[CR31] Grimm J (2000). Dissociation of Primary and Secondary Reward-Relevant Limbic Nuclei in an Animal Model of Relapse. Neuropsychopharmacology.

[CR32] Hormes JM, Kearns B, Timko CA (2014). Craving Facebook? Behavioral addiction to online social networking and its association with emotion regulation deficits. Addiction.

[CR33] Kiefer M, Marzinzik F, Weisbrod M, Scherg M, Spitzer M (1998). The time course of brain activations during response inhibition. NeuroReport.

[CR34] Kok A, Ramautar JR, De Ruiter MB, Band GPH, Ridderinkhof KR (2004). ERP components associated with successful and unsuccessful stopping in a stop-signal task. Psychophysiology.

[CR35] Kuznetsova, A., Brockhoff, P. B., & Christensen, R. H. B. (2017). lmerTest Package: Tests in Linear Mixed Effects Models. *Journal of Statistical Software*, *82*(13). 10.18637/jss.v082.i13

[CR36] Lang, P. J., Bradley, M. M., & Cuthbert, B. N. (2008). International affective picture system (IAPS): Affective ratings of pictures and instruction manual. In *Technical Report A-8.*

[CR37] LaRose R, Lin CA, Eastin MS (2003). Unregulated Internet Usage: Addiction, Habit, or Deficient Self-Regulation?. Media Psychology.

[CR38] Littel M, Franken IHA (2007). The effects of prolonged abstinence on the processing of smoking cues: an ERP study among smokers, ex-smokers and never-smokers. Journal of Psychopharmacology.

[CR39] Littel, M., van den Berg, I., Luijten, M., van Rooij, A. J., Keemink, L., & Franken, I. H. A. (2012). Error processing and response inhibition in excessive computer game players: an event-related potential study. *Addiction Biology*, *17*(5), 934–947. 10.1111/j.1369-1600.2012.00467.x 10.1111/j.1369-1600.2012.00467.x 22734609

[CR40] Logan GD, Schachar RJ, Tannock R (1997). Impulsivity and Inhibitory Control. Psychological Science.

[CR41] Lubman DI, Yücel M, Kettle JWL, Scaffidi A, MacKenzie T, Simmons JG, Allen NB (2009). Responsiveness to Drug Cues and Natural Rewards in Opiate Addiction. Archives of General Psychiatry.

[CR42] Luijten, M., Machielsen, M., Veltman, D., Hester, R., de Haan, L., & Franken, I. (2014). Systematic review of ERP and fMRI studies investigating inhibitory control and error processing in people. *Journal of Psychiatry & Neuroscience*, *39*(3), 149–169. 10.1503/jpn.130052 10.1503/jpn.130052 PMC3997601 24359877

[CR43] Marino C, Vieno A, Altoè G, Spada MM (2017). Factorial validity of the Problematic Facebook Use Scale for adolescents and young adults. Journal of Behavioral Addictions.

[CR44] Mazman SG, Usluel YK (2011). Gender differences in using social networks. Turkish Online Journal of Educational Technology.

[CR45] Moretta T, Buodo G (2018). Modeling Problematic Facebook Use: Highlighting the role of mood regulation and preference for online social interaction. Addictive Behaviors.

[CR46] Moretta T, Sarlo M, Buodo G (2019). Problematic Internet Use: The Relationship Between Resting Heart Rate Variability and Emotional Modulation of Inhibitory Control. Cyberpsychology, Behavior, and Social Networking.

[CR47] Nieuwenhuis, S., Yeung, N., van den Wildenberg, W., & Ridderinkhof, K. R. (2003). Electrophysiological correlates of anterior cingulate function in a go/no-go task: Effects of response conflict and trial type frequency. *Cognitive, Affective, & Behavioral Neuroscience*, *3*(1), 17–26. 10.3758/CABN.3.1.17 10.3758/cabn.3.1.17 12822595

[CR48] Nijs, I. M. T., Franken, I. H. A., & Muris, P. (2010). Food-related Stroop interference in obese and normal-weight individuals: Behavioral and electrophysiological indices. *Eating Behaviors*. 10.1016/j.eatbeh.2010.07.002 10.1016/j.eatbeh.2010.07.002 20850061

[CR49] Palermo, S., Stanziano, M., & Morese, R. (2018). Commentary: Anterior Cingulate Cortex and Response Conflict: Effects of Frequency, Inhibition and Errors. *Frontiers in Behavioral Neuroscience*, *12*. 10.3389/fnbeh.2018.00171 10.3389/fnbeh.2018.00171 PMC6092509 30138490

[CR50] Pastore, M., Lionetti, F., & Altoè, G. (2017). When One Shape Does Not Fit All: A Commentary Essay on the Use of Graphs in Psychological Research. *Frontiers in Psychology*, *8*. 10.3389/fpsyg.2017.01666 10.3389/fpsyg.2017.01666 PMC5622191 28993749

[CR51] Patton JH, Stanford MS, Barratt ES (1995). Factor structure of the barratt impulsiveness scale. Journal of Clinical Psychology.

[CR52] Porjesz, B., & Begleiter, H. (2003). Alcoholism and human electrophysiology. In *Alcohol Research and Health*. PMC6668890 15303626

[CR53] Potenza MN (2006). Should addictive disorders include non-substance-related conditions?. Addiction.

[CR54] R Development Core Team. (2016). R: A language and environment for statistical computing. In *R Foundation for Statistical Computing*.

[CR55] Ramautar JR, Kok A, Ridderinkhof KR (2004). Effects of stop-signal probability in the stop-signal paradigm: The N2/P3 complex further validated. Brain and Cognition.

[CR56] Roche RAP, Garavan H, Foxe JJ, O’Mara SM (2005). Individual differences discriminate event-related potentials but not performance during response inhibition. Experimental Brain Research.

[CR57] Rothen S, Briefer J-F, Deleuze J, Karila L, Andreassen CS, Achab S, Thorens G, Khazaal Y, Zullino D, Billieux J (2018). Disentangling the role of users’ preferences and impulsivity traits in problematic Facebook use. PLOS ONE.

[CR58] Schulz KP, Fan J, Magidina O, Marks DJ, Hahn B, Halperin JM (2007). Does the emotional go/no-go task really measure behavioral inhibition?Convergence with measures on a non-emotional analog. Archives of Clinical Neuropsychology.

[CR59] Sokhadze E, Stewart C, Hollifield M, Tasman A (2008). Event-Related Potential Study of Executive Dysfunctions in a Speeded Reaction Task in Cocaine Addiction. Journal of Neurotherapy.

[CR60] Spada MM, Marino C (2017). Metacognitions and emotion regulation as predictors of problematic internet use in adolescents. Clinical Neuropsychiatry.

[CR61] Sun D-L, Chen Z-J, Ma N, Zhang X-C, Fu X-M, Zhang D-R (2009). Decision-Making and Prepotent Response Inhibition Functions in Excessive Internet Users. CNS Spectrums.

[CR62] Wagenmakers E-J, Farrell S (2004). AIC model selection using Akaike weights. Psychonomic Bulletin & Review.

[CR63] Wang H-Y, Sigerson L, Cheng C (2019). Digital Nativity and Information Technology Addiction: Age cohort versus individual difference approaches. Computers in Human Behavior.

[CR64] Wölfling K, Mörsen CP, Duven E, Albrecht U, Grüsser SM, Flor H (2011). To gamble or not to gamble: At risk for craving and relapse – learned motivated attention in pathological gambling. Biological Psychology.

[CR65] Yu JJ, Kim H, Hay I (2013). Understanding adolescents’ problematic Internet use from a social/cognitive and addiction research framework. Computers in Human Behavior.

[CR66] Zhang W, Lu J (2012). Time course of automatic emotion regulation during a facial Go/Nogo task. Biological Psychology.

[CR67] Zhou Z-H, Yuan G-Z, Yao J-J, Li C, Cheng Z-H (2010). An event-related potential investigation of deficient inhibitory control in individuals with pathological Internet use. Acta Neuropsychiatrica.

